# Tramadol Induced Adrenal Insufficiency: Histological, Immunohistochemical, Ultrastructural, and Biochemical Genetic Experimental Study

**DOI:** 10.1155/2017/9815853

**Published:** 2017-11-27

**Authors:** Shereen Abdelhakim Abdelaleem, Osama A. Hassan, Rasha F. Ahmed, Nagwa M. Zenhom, Rehab A. Rifaai, Nashwa F. El-Tahawy

**Affiliations:** ^1^Forensic Medicine & Toxicology Department, Faculty of Medicine, Minia University, Minia, Egypt; ^2^Biochemistry Department, Faculty of Medicine, Minia University, Minia, Egypt; ^3^Histology Department, Faculty of Medicine, Minia University, Minia, Egypt

## Abstract

Tramadol is a synthetic, centrally acting analgesic. It is the most consumed narcotic drug that is prescribed in the world. Tramadol abuse has dramatically increased in Egypt. Long term use of tramadol can induce endocrinopathy. So, the aim of this study was to analyze the adrenal insufficiency induced by long term use of tramadol in experimental animals and also to assess its withdrawal effects through histopathological and biochemical genetic study. Forty male albino rats were used in this study. The rats were divided into 4 groups (control group, tramadol-treated group, and withdrawal groups). Tramadol was given to albino rats at a dose of 80 mg/kg body weight for 3 months and after withdrawal periods (7–15 days) rats were sacrificed. Long term use of tramadol induced severe histopathological changes in adrenal glands. Tramadol decreased the levels of serum cortisol and DHEAS hormones. In addition, it increased the level of adrenal MDA and decreased the genetic expression of glutathione peroxidase and thioredoxin reductase in adrenal gland tissues. All these changes started to return to normal after withdrawal of tramadol. Thus, it was confirmed that long term use of tramadol can induce severe adrenal insufficiency.

## 1. Introduction

Tramadol Hydrochloride (Tramal™) is a synthetic centrally acting analgesic drug with opioid and nonopioid properties. It is used parenterally and orally for the treatment of moderate to severe pain due to its relatively lower risk of respiratory depression or physical dependence and better safety profile when compared with other opiates [1].

The molecular formula of tramadol is C_16_H_25_NO_2_. It is rapidly absorbed after oral administration and its peak blood level is achieved about 2-3 hours thereafter. Its half-life is about 5-6 hours and it is metabolized in liver by demethylation. Its active metabolite (O-desmethyltramadol) has a higher affinity for the mu-opioid receptors and twice the analgesic effect of the parent drug [2].

The most common adverse effects of tramadol are nausea, vomiting, dizziness, anorexia, seizures, and hypotension which may occur in therapeutic or toxic doses [3]. The most common mechanisms of death after tramadol overdose are cardiorespiratory depression, resistant shock, asystole, and liver failure. Fatal toxicity of tramadol has been reported after coadministration of other medications including propranolol, ethanol, barbiturates, and benzodiazepines [4].

Recently, young adult addicts “typically substituted tramadol for heroin.” Repeated tramadol administration in such patients might lead to accumulation of toxic metabolites in their blood and thus increase its potential for toxicity [5]. Tramadol abuse has dramatically increased in Egypt since 2008 and has led to many serious health and social problems and to many admissions to addiction treatment centers [6].

There is a recent study that revealed that about 40% of temporary cleaners and 21% of permanent cleaners working in governmental hospitals in Zagazig city in Egypt used tramadol. A recent governmental investigation of 1800 truck and microbus drivers found that 200 (11%) of them admitted to regular usage of drugs when working. Also, tramadol abuse is associated with 18.7% of death rates due to road traffic accidents [7].

Many researchers have explored and evaluated the effects of chronic use of tramadol on many organs, for example, liver, kidney, testes, heart, and thyroid gland. Owing to the few number and scanty data in the evaluation of the effects of chronic use of tramadol on the endocrine system, the objective of this study was to analyze the adrenal insufficiency induced in albino rats by chronic use of tramadol and its withdrawal effects.

## 2. Materials and Methods

### 2.1. Animals

This study was carried out on 40 adult male Wistar albino rats (weighing approximately 200–250 gm body weight) of 12 weeks of age. These rats were obtained from Minia University laboratory animals growing center in Minia, Egypt. They were housed in plastic cages and fed a standard laboratory diet (ad libitum) and water during the experimental period. All rats were kept under good ventilation and aerated room at a laboratory temperature of 22 ± 3°C. All aspects of animal care and treatment were carried out according to the local guide lines of the ethical committee of Faculty of Medicine, Minia University, Egypt.

### 2.2. Chemicals

Tramal (Tramadol HCL), 50 mg tablets, was obtained from Memphis for Pharmaceutical and Chemical Industries, Egypt. It was dissolved in water and according to the study of “Matthiesen et al. [8]” the LD_50_ values of oral administration of tramadol were estimated to be about 300–350 mg/Kgm body weight for rats and mice. Tramadol was given at a dose of 80 mg/Kgm body weight according to “El-Gaafarawi [9].” This therapeutic dose was calculated according to “Paget and Barnes [10].” The chosen dose was nearly comparable to the human effective therapeutic dose.

### 2.3. Experimental Work

This experimental work was done in the departments of Forensic Medicine & Clinical Toxicology, Biochemistry, and Histology, in Faculty of Medicine, Minia University, Egypt, during the period from 1 January to the end of March 2017. Animals were divided into 4 groups (10 rats for each group) as follows:

  Group I (control group): the rats were fed a standard laboratory diet and water
  Group II (tramadol-treated group): the rats were given tramadol at a dose of 80 mg/Kgm body weight orally through nasogastric tube for 12 weeks
  Groups III and IV: they were the withdrawal tramadol groups. Rats that received tramadol were sacrificed after one week of tramadol withdrawal in group 3 and after 2 weeks of tramadol withdrawal in group 4.

Rats were sacrificed by decapitation after light ether anesthesia and dissected at the end of 12th, 13th, and 14th weeks of treatment. All approved conditions used for animal housing and handling were considered. The used experimental protocol followed the regulations for administration and painless sacrifice of the experimental animals.

### 2.4. Histological Studies

#### 2.4.1. Light Microscopic Examinations

Parts of each adrenal gland were removed intact and were fixed in 10% buffered formalin followed by paraffin embedding using routine procedures. Seven-micrometer sections were deparaffinized with xylene and stained with haematoxylin and eosin (H&E) to be viewed by light microscopy to examine the adrenal glands [11].

#### 2.4.2. Immunohistochemical Studies

Other sections were mounted on poly-L-Lysine coated slides and used for immunohistochemical staining with anti-iNOS (inducible nitric oxide synthase) and anti-Caspase-8 according to the previously published protocol [12]. Briefly, sections were deparaffinized and rehydrated, and, in order to retrieve antigen, sections were incubated with 0.1% trypsin and 0.1% CaCl_2_ 2H_2_O (calcium chloride dehydrate) in 50 mM Tris (Trisaminomethane) buffered saline at pH 7.4 at 37°C for 120 minutes. Sections were soaked in absolute methanol containing 0.3% hydrogen peroxide for 30 min at room temperature to eliminate the endogenous peroxidase activity. The sections were then incubated with 1.5% nonimmunized goat serum for 30 min at room temperature and then incubated with diluted iNOS and diluted Caspase-8 (1 : 500) for 30 min and washed three times with phosphate buffer saline (PBS). Thereafter, the sections were incubated with biotinylated goat anti-mouse Ig serum for 60 min. After being washed with PBS, the sections were incubated with avidin/biotin peroxidase complex. Sites of peroxidase binding were detected using chromogenic 3,3-diaminobenzidine (DAB) tetrahydrochloride substrate. Tissue sections were counterstained with haematoxylin [12].

Tissue sections (adrenal gland) were examined and images were digitally captured using a hardware consisting of high-resolution color digital camera mounted on an Olympus microscope (Olympus BX51, Tokyo, Japan) and connected to a computer and then dealt with using adobe Photoshop.

#### 2.4.3. Electron Microscopic Examinations

Tissues were removed and placed in 2.7% glutaraldehyde-0.1 ml phosphate buffer solution for 2 hr and then flooded in 4 consecutive baths of the same fixative 0.15 ml phosphate buffer (4°C) for 1 hr each. The samples were postfixed in a 2% osmic acid-0.15 ml phosphate buffer solution (4°C) for 1 hr, and again 4 times flooded in 0.15 ml phosphate buffer solution for 15 min each. Samples were acetone dehydrated and embedded in polyesteric resin, polymerized at 60°C for 72 hrs (Semithin sections (1 *μ*m) stained with toluidine blue). Ultrathin sections were made using ultramicrotome and double contrasted with uranyl acetate and lead citrate solutions [13] and examined by JEOL electron microscope (JEM-100CXII) equipped with a camera.

### 2.5. Biochemical Studies

At the end of the experiment, blood samples were withdrawn from the retroorbital plexus of veins using a capillary pipette and collected in heparinized tubes containing 5000 I.U/ml heparin Sodium and centrifuged at 3000 rPm (revolutions per minute) for 15 min. Plasma was separated and stored at −20°C until required. Each sample was used to evaluate the following levels:

- Cortisol level: cortisol ELISA kits were obtained from antibodies-online GmbH Company, Egypt, according to “Herrero et al. [14]”. The measurement unit is nmol/L.
- DHEAS (dehydroepiandrosterone sulfate) level: the ELISA kits were obtained from GenWay Biotech Company, Egypt, according to “Grimby-Ekman et al. [15].” The measurement unit is microgram/ml.
- Glutathione peroxidase (GSH-Px) level: the kits were obtained from Biodiagnostic Company, Egypt, colorimetric method [16]. The unit of measurement is mIU/ml.
- Thioredoxin reductase (TR) level: the kits were obtained from Biodiagnostic Company, Egypt, colorimetric method [17]. The unit of measurement is mIU/ml.

After scarification of rats, parts of adrenal glands were dissected and fixed in 10% formalin and 2.7% glutaraldehyde for histological, immunohistochemical, and ultrastructural studies. The remaining adrenal glands were divided into 2 parts. The 1st part was frozen in liquid nitrogen and stored at −80°C until the analysis of GSH-Px and TR mRNA expression by RT-PCR. The 2nd part was used for biochemical analysis of malondialdehyde (MDA). The kits of MDA were obtained from Biodiagnostic Company, Egypt, and the method of its estimation was done according to “Ohkawa et al. [18].” The measurement unit is *μ*mol/mg wet tissue.

*(i) GSH-Px and TR mRNA Expression Quantified by Reverse Transcription (RT-) Polymerase Chain Reaction (PCR)*. Total RNA was purified from homogenized suprarenal gland specimen using Ribozol RNA Extraction Reagent (Amresco, Solon, USA) following the manufacturer's instructions. The purity and integrity of the total RNA were determined by spectrophotometry and gel electrophoresis. Reverse transcriptase polymerase chain reaction (RT-PCR) was performed with Qiagen one-step RT-PCR kit (Qiagen, Germany) as per the manufacturer's instructions; amplification was performed in a thermal cycler (Progene; Techne Ltd., Duxford, United Kingdom).

Primers, amplicon size, and annealing temperature of glutathione peroxidase (GPX), thioredoxin reductase, B-actin (as internal control for GSH-PX), and 18S (an internal control for TR) were demonstrated in Table 1. The RT-PCR conditions were as the follows: (a) reverse transcription, 30 min, 50°C. (b) Initial PCR activation step, 15 min, 95°C. (c) 3-step cycling for 30 cycles, each cycle consisting of denaturation for 30 sec at 94°C followed by annealing for 30 sec at temperature as in Table 1 and extension for 1 min at 72°C. RT-PCR products were separated on 2% agarose gel, visualized by ethidium bromide staining [19, 20]. The intensity of the PCR product bands was quantified using gel documentation system software (Biometra GmbH, Germany).

### 2.6. Statistical Analysis

Values were expressed as mean ± standard deviation (SD). The data were analyzed by using SPSS for windows (version 10.0). The significance of differences was calculated by using One-Way ANOVA test. *P* < 0.05 was considered significant.

## 3. Results

### 3.1. Results of Histological Studies

**Figure 1:**
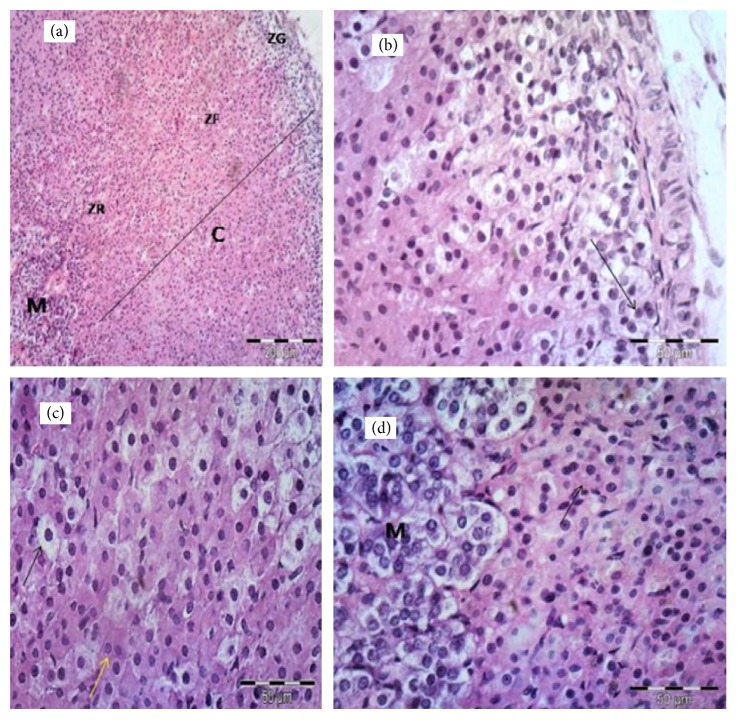
Photomicrographs of the adrenal gland of the control group showing (a) adrenal cortex (C), zona glomerulosa (ZG), zona fasciculata (ZF), zona reticularis (ZR), and adrenal medulla (M) ×100. (b) A higher magnification of ZG cells arranged in oval or rounded clusters and with deeply stained nuclei (arrow), ×400. (c) A higher magnification of ZF cells showing highly vacuolated cells (black arrow) and less vacuolated cells with acidophilic cytoplasm (yellow arrow) and vesicular nuclei, ×400. (d) A higher magnification of ZR showing small cells with deeply acidophilic cytoplasm (arrow); M, medulla, ×400.

Figure 1 shows that the suprarenal gland of group I was formed of two parts, the adrenal cortex and the adrenal medulla. The cortex displayed three zones, zona glomerulosa (ZG), zona fasciculata (ZF), and zona reticularis (ZR) (Figure 1(a)). Cells of ZG layer form oval or rounded clusters with deeply stained nuclei (Figure 1(b)). Cells of zona fasciculata (ZF) are arranged in straight cords separated by capillaries. Some cells were large and polyhedral with pale vacuolated cytoplasm. Others appeared less vacuolated with acidophilic cytoplasm and vesicular rounded nuclei (Figure 1(c)). Cells of ZR were small and deeply stained and form irregular cords and clusters, separated by capillaries. The adrenal medulla was at the center. Its cells were rounded in shape, rather basophilic, and arranged in cluster (Figure 1(d)).

**Figure 2:**
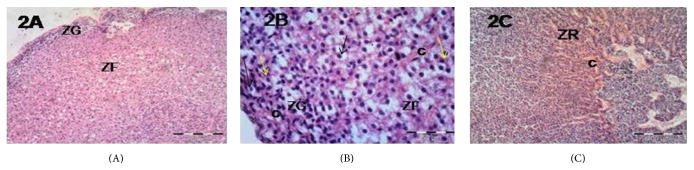
Photomicrographs of the adrenal gland of group II showing (A) loss of normal architecture of ZG and ZF, ×100; (B) higher magnification of ZG and ZF showing some cells with marked vacuolated cytoplasm (black arrow) and pyknotic nuclei; others are shrunken (yellow arrow) with pyknotic nuclei and deeply acidophilic cytoplasm; notice the congested blood capillaries (c), ×400; (C) ZR with markedly congested blood capillaries (c), ×100.

**Figure 3:**
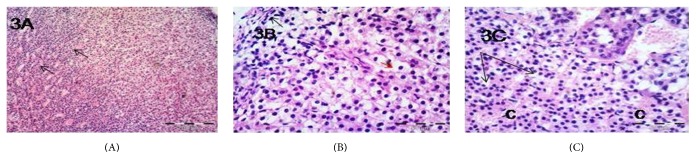
Photomicrographs of the adrenal gland of group III showing (A) marked congested blood capillaries of ZF and ZR (arrows), ×100; (B) cells with marked vacuolated cytoplasm and pyknotic nuclei in ZG (black arrows) and ZF (red arrows), ×400; (C) ZR showing cells with vacuolated cytoplasm and pyknotic nuclei (arrows) and markedly congested capillaries (c), ×400.

**Figure 4:**
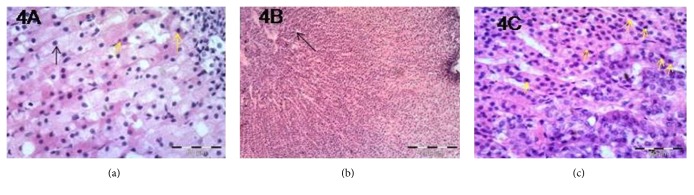
Photomicrographs of the adrenal gland of group IV showing (a) ZF cells appearing nearly similar to that of control (black arrow); few cells appear shrunken with hyperacidophilic cytoplasm and pyknotic nuclei (yellow arrows), ×400 (b); congested blood capillaries especially at the area of ZR (arrow), ×100; (c) numerous mitotic figures (arrows) in ZR, ×400.

Examination of sections of the adrenal cortex of group II revealed loss of normal architecture of the ZG and ZF (Figure 2(a)). Most of the ZG and ZF cells appeared ballooned and vacuolated. Their nuclei became pyknotic. Some cells were shrunken with pyknotic nuclei (Figure 2(b)). The blood capillaries of ZG, ZF, and ZR were markedly congested (Figures 2(b) and 2(c)). The same morphological changes were observed in group III as that in group II (Figures 3(a), 3(b), and 3(c)). Examination of sections from group IV revealed that most of the morphological changes observed in groups II and III were improved. Many ZF cells were more or less similar to that of the control group. Still some ZF cells were shrunken with hyperacidophilic cytoplasm and pyknotic nuclei (Figure 4(a)). Many congested blood capillaries were observed specially in ZR (Figure 4(b)). Interestingly many mitotic figures were observed in the cells of ZR (Figure 4(c))

**Figure 5:**
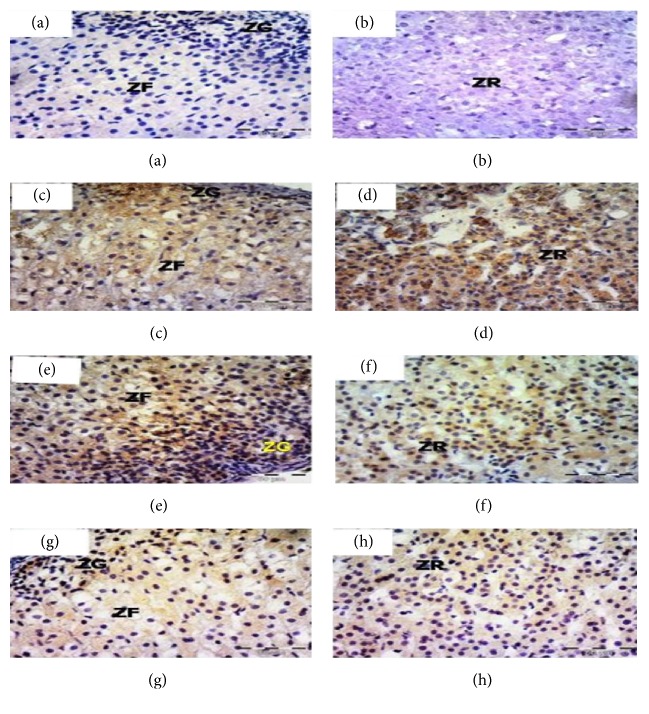
Rat adrenal cortex labeled for iNOS of the control group (a and b) showing negative expression; Group II (c and d) showing high expression along the adrenal cortex; Group III showing high expression in ZG and ZR (e) and moderate expression in ZR (f); Group IV (g and h) showing faint expressions through the adrenal cortex, ×400. Zona glomerulosa (ZG), zona fasciculata (ZF), and zona reticularis (ZR).

Immunohistochemical staining for iNOS revealed that the sections of the control group showed negative expression along the length of the adrenal cortex including ZG, ZF, and ZR (Figures 5(a) and 5(b)). High positive expression was noticed in the cytoplasm of cells of all zones of the adrenal cortex in group II (Figures 5(c) and 5(d)). After one week of withdrawal, group III, the expression was high in ZG and ZF (Figure 5(e)), while in ZR moderate expression was noticed (Figure 5(f)). In group IV, faint expression was observed in the cytoplasm of the ZG, ZF, and ZR cells (Figures 5(g) and 5(h)).

**Figure 6:**
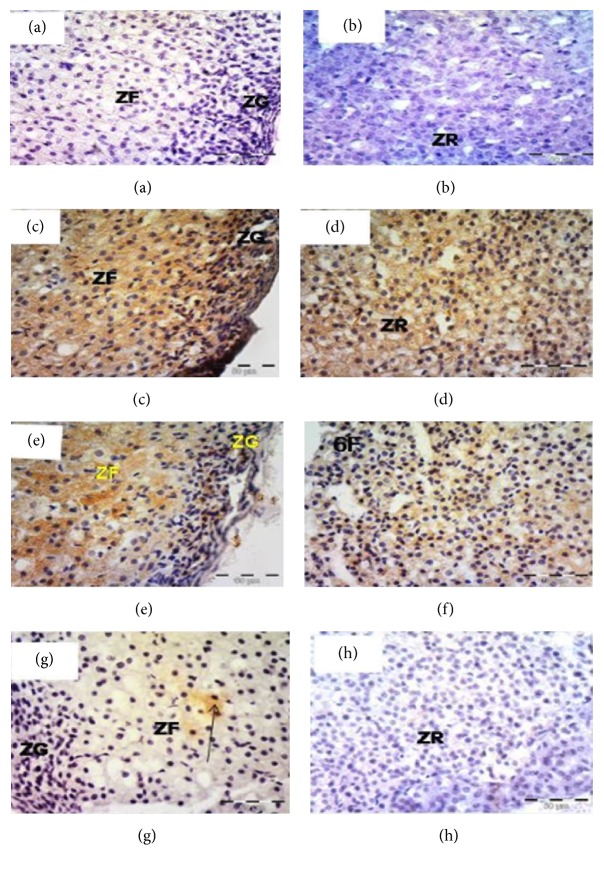
Rat adrenal cortex labeled for caspase-3 of the control group (a and b) showing negative expression; Group II (c and d) showing high expression along the adrenal cortex; Group III showing high expression in ZG and ZR (e) and moderate expression in ZR (f); Group IV (g and h) showing few scattered immunopositive cells among ZF (arrow), ×400. Zona glomerulosa (ZG), zona fasciculata (ZF), and zona reticularis (ZR).

The caspase-8 immunostaining was negative in the control group along the adrenal cortex (Figures 6(a) and 6(b)). Dense expression was noticed in almost all glomerulosa, fasciculata, and reticularis cellular cytoplasm (Figures 6(c) and 6(d)). In group III, the expression was moderate in cells of ZG and ZF (Figure 6(e)), while cells of ZR showed faint cytoplasmic expression (Figure 6(f)). Sections from group IV showed scattered immunopositive cells among ZF (Figure 6(g)), while those of ZG and ZF cells appeared negative (Figure 6(h)).

**Figure 7:**
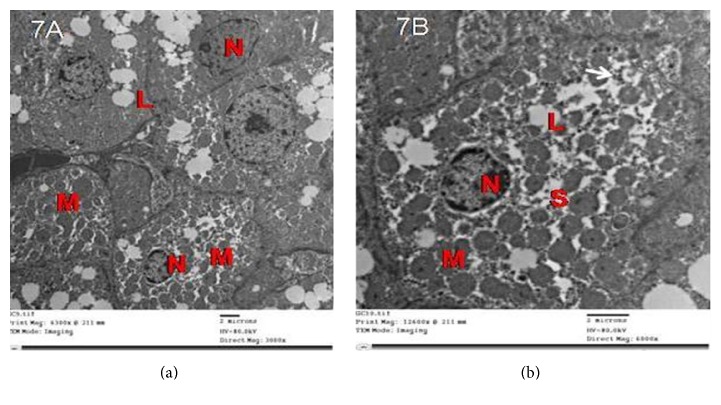
Electron micrographs of the adrenal cortex of a control group showing ZG cells with (a) spherical or irregular nuclei (N) with peripheral heterochromatin, numerous mitochondria (M), and few lipid droplets (L). (b) Irregular nuclei (N) with peripheral heterochromatin, many mitochondria (M), smooth endoplasmic reticulum (S), some lipid droplets (L), and free ribosomes (arrow).

Results of electron microscopic examinations (EM) revealed that the adrenal cortex of a control rat showed cells of the zona glomerulosa (ZG) with numerous mitochondria, free ribosomes, and few lipid droplets. The lipid droplets were seen as electron lucent with distinct boundaries. The nucleus was spherical or irregular and euchromatic with peripheral heterochromatin (Figure 7).

**Figure 8:**
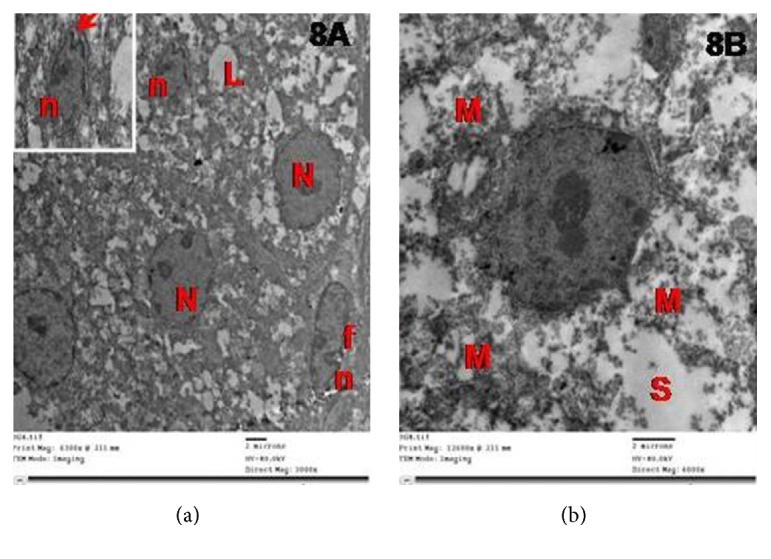
Electron micrographs of the adrenal cortex in group II showing that ZG cells with (a) nuclei (N) have irregular outlines and few lipid droplets (L). Notice the shrunken nucleus (n) with widened perinuclear space (arrow in inset). Notice the fibroblast of the capsule with elongated nucleus (fn). (b) Degenerated mitochondria with disrupted cristae (M) and dilated cisternae of SER (s).

ZG cells in group II showed numerous degenerated mitochondria with disrupted cristae, dilated cisternae of SER, and few lipid droplets. The nuclei had irregular outlines, shrunken with widened perinuclear space of the nuclear envelope (Figure 8).

**Figure 9:**
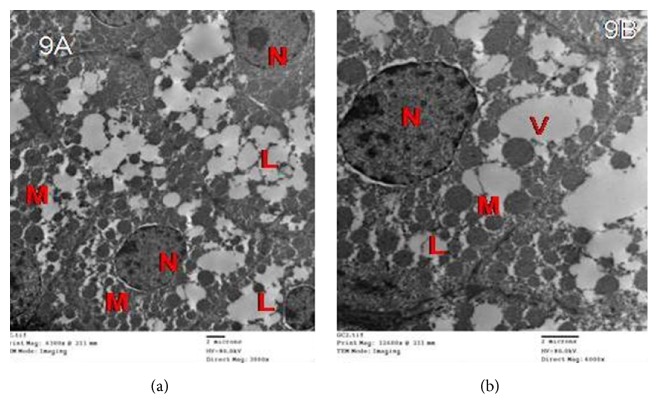
Electron micrographs of the adrenal cortex in group III showing that ZG cells with (a) euchromatic nuclei (N) have irregular outlines, an apparent increase in lipid droplets (L), and mitochondria with intact cristae (M). (b) showings cytoplasmic vacuoles (V) and a euchromatic nucleus with peripheral clumps of heterochromatin (N). Note: numerous lipid droplets and mitochondria (M) more or less as the control.

The tramadol withdrawal for 1 week showed some enhancement as the adrenal cortex in group III showed ZG cells with euchromatic nuclei of irregular outlines and peripheral clumps of heterochromatin, an apparent increase in lipid droplets, and mitochondria with intact cristae more or less as the control. Some cells showed lipid droplets of indistinct boundaries; widened perinuclear space and large vacuoles were also observed (Figure 9).

**Figure 10:**
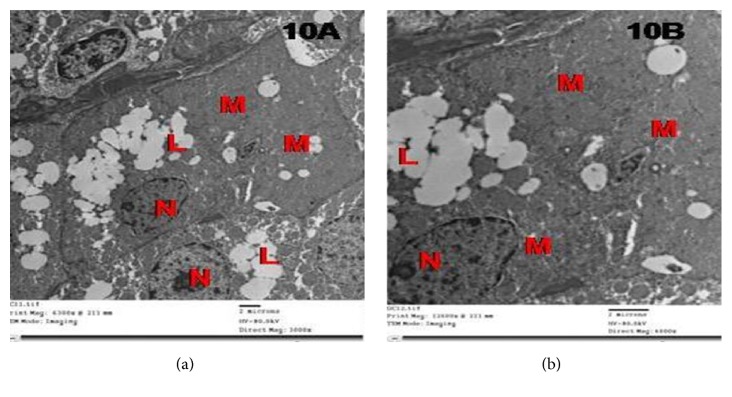
Electron micrographs of the adrenal cortex in group IV showing ZG cells showing euchromatic nuclei with peripheral clumps of heterochromatin (N). Note numerous normal mitochondria (M) and decreased lipid droplets compared to group III.

While tramadol withdrawal for 2 weeks showed more enhancement of the adrenal cortex in group IV as ZG cells had euchromatic nuclei with peripheral clumps of heterochromatin, numerous normal mitochondria with intact cristae and decreased lipid droplets compared to group III (Figure 10).

**Figure 11:**
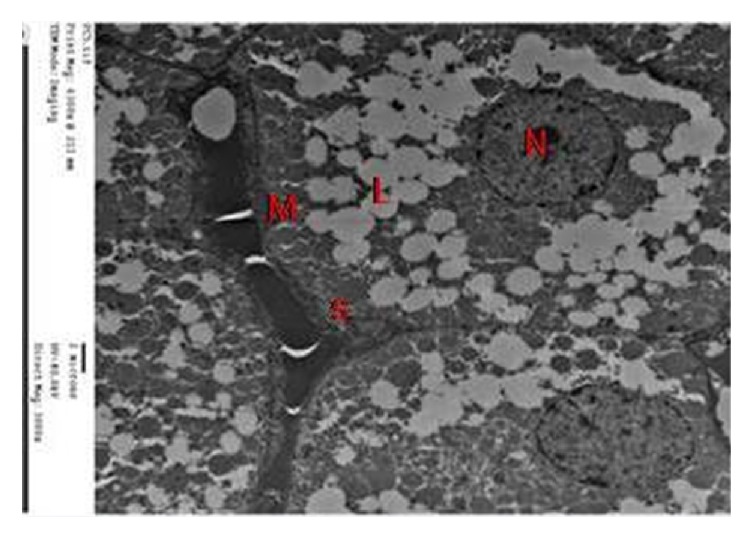
An electromicrograph of control zona fasciculata cells showing many mitochondria with vesicular cristae (M), smooth endoplasmic reticulum (S), lipid droplets (L), and a euchromatic nucleus with peripheral clumps of heterochromatin (N).

By EM, zona fasciculata cells of group I showed numerous lipid droplets, many mitochondria with vesicular cristae, smooth endoplasmic reticulum, and euchromatic nucleus with peripheral clumps of heterochromatin (Figure 11).

**Figure 12:**
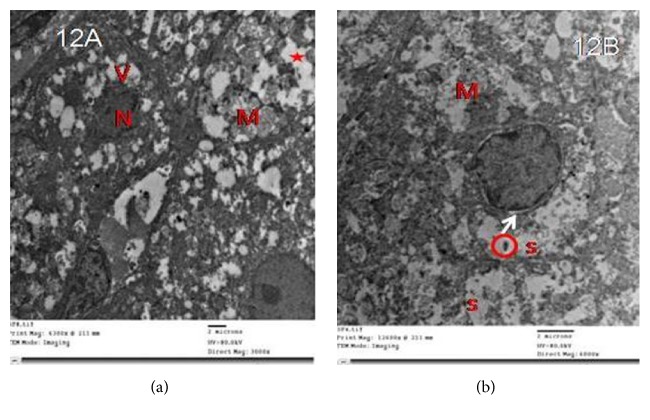
(a) Electron micrographs of zona fasciculata cells of group II showing vacuoles of variable sizes (V), mitochondria with destroyed cristae (M), lipid droplets without discernible outline (L), and a shrunken hyperchromatic nucleus with an irregular outline (N). Note confluent lipid droplets (star). (b) Another cell has an irregular nucleus (N) with dilated perinuclear space (arrow), mitochondria with destroyed cristae (M), and numerous dilated profiles of smooth endoplasmic reticulum (s). Notice cytoplasmic vacuoles with electron-dense cores (circles).

Zona fasciculata cells of group II showed vacuoles of variable sizes with electron-dense cores, mitochondria with destroyed cristae, many lipid droplets without discernible outline, and a shrunken hyperchromatic nucleus with an irregular outline. Some of the lipid droplets fused with each other and became confluent. Other cells had irregular nuclei with dilated perinuclear space and numerous dilated profiles of smooth endoplasmic reticulum (Figure 12).

**Figure 13:**
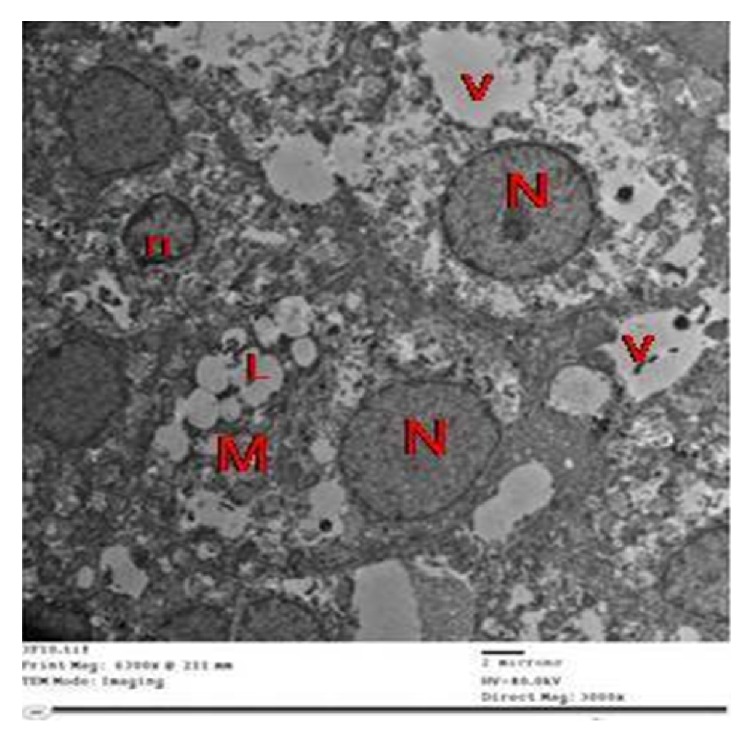
An electron micrograph showing fasciculata cells from group III containing numerous mitochondria (M) and lipid droplets (L). Note large vacuoles (V). Cells have rounded euchromatic nucleus (N) and the other has irregular dense nucleus (n).

Fasciculata cells from group III contained numerous intact mitochondria and lipid droplets. But some of the lipid droplets were still confluent and showed no discernible outline. Some cells had rounded euchromatic nuclei and others had irregular nuclei and dilated nuclear envelope (Figure 13).

**Figure 14:**
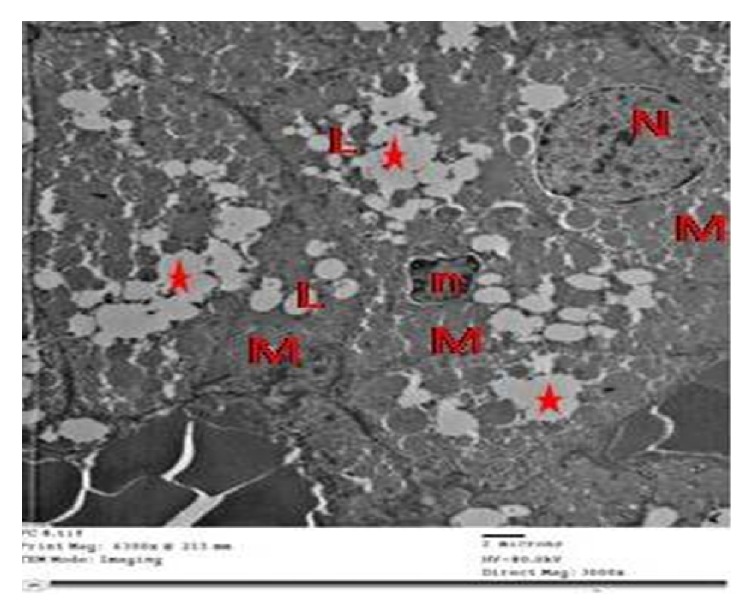
An electron micrograph showing fasciculata cells from group IV showing cells with euchromatic nuclei (N) with regular outline (arrow), numerous mitochondria with intact cristae (M), and numerous lipid droplets (L). Note some confluent lipid droplets (stars) and the irregular shrunken hyperchromatic nucleus (n).

Fasciculata cells from group IV showed most cells with euchromatic nuclei with regular outline, numerous mitochondria with intact cristae, and numerous lipid droplets. Few cells had confluent lipid droplets and scarce nuclei were shrunken and hyperchromatic (Figure 14).

### 3.2. Results of Biochemical Studies

Cortisol and DHEAS levels showed a significant decrease in groups II, III, and IV when compared with group I. Also, there is a significant increase in both hormonal levels in groups III and IV compared to group II. Two-week withdrawal of tramadol in group IV resulted in significant increase in the levels of the previous hormones than group III (one-week withdrawal of tramadol) but still did not reach its level in control group (Table 2).

Tables 3 and 4 showed that there was a significant decrease in GSH-Px and TR, respectively, in groups II, III, and IV when compared to group I. Both antioxidants were significantly increased in groups III and IV compared to group II. Significant increase in both antioxidants levels was noticed in group IV compared to group III but was still below the normal level.

Adrenal gland MDA revealed significant increase in groups II, III, and IV compared to group I. MDA level was significantly decreased in groups III and IV than group II. Significant decrease in MDA level was noticed in group IV when compared to group III but still did not reach the level of the control group (Table 5).

**Figure 15:**
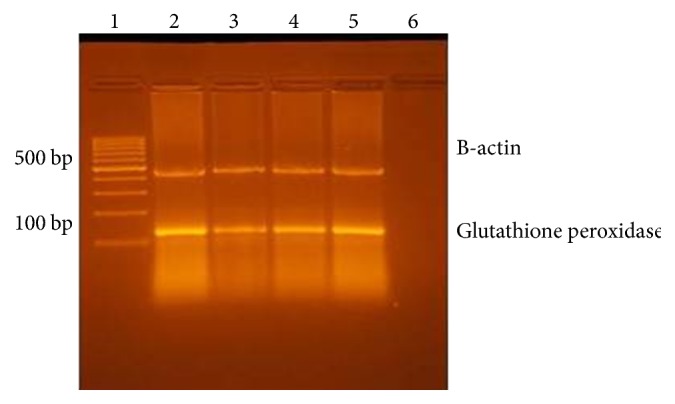
Agarose gel electrophoresis of RT-PCR products showed increase in the expression of GSH-Px genes in adrenal glands in tramadol withdrawal groups (groups III and IV) more than tramadol group (group II).

**Figure 16:**
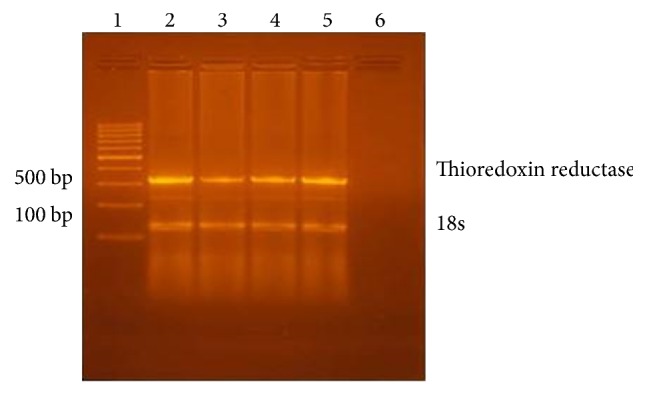
Agarose gel electrophoresis of RT-PCR products showed increase in the expression of TR genes in adrenal glands in tramadol withdrawal groups (groups III and IV) more than tramadol group (group II).

Tables 6 and 7 revealed significant decrease in the intensity of PCR product bands of GSH-Px and TR in groups II, III, and IV compared to group I. The intensity of bands was significantly increased in groups III and IV but more in group IV when it was compared to group II. Agarose gel electrophoresis of RT-PCR products showed increase in the expression of GSH-Px and TR genes in adrenal glands in tramadol withdrawal groups (groups III and IV) more than tramadol group (group II) (Figures 15 and 16).

## 4. Discussion

Tramadol is a synthetic-4-phenyl-piperidine analogue of codeine. It is a central analgesic with a low affinity for opioid receptors. It is used to treat moderate to severe pain through combination of mu-opioid agonist effects and norepinephrine and serotonin reuptake inhibition. The M1 metabolite of tramadol produced by liver-O-demethylation shows a higher affinity for opioid receptors than the parent drug [21].

Tramadol abuse has dramatically increased in Middle East region especially in Iran and Egypt. The prevalence was higher in males (77.2%). Among Secondary School male students, 0.84% abused opiates. The last national survey report stated that 9.6% of Egyptians used drugs at least once during their lives [22]. In other studies, university students with previous history of cigarette smoking and consumption of addicting opioids were liable to abuse tramadol. It has been said that, almost 90% of tramadol toxicities are acute, 7.9% are chronic, and 2.1% are acute on top of chronic overdoses [23].

Connor et al. [24] stated that the recommended daily dose of tramadol is 50–100 mg/4–6 hours and the maximum total daily dose should not exceed 400 mg secondary to the increased risk of side effects with higher doses. The LD_50_ of tramadol in mice and rats has been determined to be 350 mg/Kg body weight and 228 mg/Kg body weight, respectively, following an oral dose. In addition, an LD_50_ of 200 mg/Kg body weight and 286 mg/Kg body weight has been reported in mice and rats, respectively, following a subcutaneous dose [8].

Tramadol in this present study was given at a dose of 80 mg/Kg body weight orally (35% of oral LD_50_ of tramadol in rats) according to the study of “El-Gaafarawi [9].” Severe pathological changes were observed in adrenal glands of rats, although none of the rats died during the experimental period.

Opioid-induced endocrinopathy is one of the most common, yet, least often diagnosed consequences of prolonged opioid therapy. Many studies have evaluated the effects of chronic use of tramadol on many organs, for example, liver, kidney, testes, ovaries, heart, and thyroid gland. Owing to the presence of limited numbers of literatures that study the pathological effects of tramadol on adrenal glands, the authors ran out this work to illustrate the dramatic histopathological effects of tramadol on adrenal glands.

The present study proved that chronic use of tramadol induced pathological changes in adrenal glands in the form of loss of normal architecture of ZF and ZG. The cells became ballooned and vacuolated with pyknotic nuclei and congested blood capillaries by light microscopic examination. Immunohistochemical staining for iNOS and Caspase-8 revealed high positive expression in the cytoplasm of cells of all zones of adrenal cortex of rats in tramadol-treated group.

Ultrastructural examinations of adrenal glands in this present study showed numerous degenerated mitochondria with disrupted cristae and irregular outlines of nuclei of ZG cells. ZF cells showed multiple vacuoles, shrunken hyperchromatic nuclei with an irregular outline, and dilated profiles of smooth endoplasmic reticulum.

The pathological changes and oxidative damage induced by chronic use of tramadol can be explained by its capability to generate oxygen free radicals that can attack the cell membrane and lead to destabilization and disintegration of cell membrane as a result of lipid peroxidation [25].

After one-week withdrawal of chronic tramadol use, the same morphological changes were observed in group III as that in group II by light microscopic examination. By immunohistochemical study, the expression of iNOS and Caspase-8 was high in ZG and ZF, while, in ZR, moderate expression was noticed. Ultrastructural examination of adrenal glands revealed some enhancement of ZG and ZF cells and appearance of euchromatic nuclei of irregular outline and peripheral clumps of heterochromatin.

Two-week withdrawal of tramadol led to more improvement of the morphological changes in ZG, ZF, and ZR cells more or less similar to that of control group by light and electron microscopic examination. Faint expression of iNOs and Caspase-8 in the cytoplasm of ZG, ZF, and ZR cells was noticed by immunohistochemical studies.

To our knowledge, after surfing all available search engines, there is no single study that could determine the biochemical oxidative stress induced by chronic tramadol use and its withdrawal effects on adrenal gland and the effect of tramadol on the genetic expression of some antioxidant enzymes; therefore, the aim of this study was to determine the biochemical changes (hormonal, oxidative stress, and genetic expression) that can be induced by chronic use of tramadol and its withdrawal.

Chronic tramadol use in the research led to significant increase in the level of adrenal MDA, in addition to a significant decrease in the level of antioxidant enzymes (GSH-Px and TR) in the blood. Tramadol withdrawal resulted in significant decrease in the level of MDA and significant increase in the level of the above enzymes after 2-week withdrawal was more than one-week tramadol withdrawal. These results were confirmed by studying the genetic expression of GSH-Px and TR in suprarenal gland. Agarose gel electrophoresis of RT-PCR products showed increase in the expression of GSH-Px and TR genes in adrenal glands in tramadol withdrawal groups (groups III and IV) more than tramadol group (group II).

Ghoneim et al. [26] and Nna and Osim [27] studied the oxidative stress markers during and after withdrawal of tramadol administration. Their study revealed that chronic tramadol use increased the level of MDA and decreased the level of catalase, superoxide dismutase, and glutathione peroxidase in both testicular and brain tissues and improvement of these markers occurred after tramadol withdrawal.

The hormonal changes induced by chronic tramadol use in this study revealed a significant decrease in the levels of cortisol and DHEAS in tramadol group and a significant increase in their level after tramadol withdrawal (more after 2-week tramadol withdrawal). These results cope with the results as discussed by other studies [28–31]. Their study revealed that chronic use of exogenous opioids has been found to decrease ACTH and cortisol levels. In addition, their study revealed that levels of DHEAS, a precursor of adrenal androgens, have also been decreased in chronic opioid users.

Knowledge of opioid-induced adrenal insufficiency is limited by the lack of large scale studies. Opioids may decrease adrenal stress response, leading to symptoms of adrenal insufficiency during acute illness or stress. The main important mechanism in opioid-induced endocrinopathy is the large dramatic effect of opioids on the hypothalamic-pituitary-adrenal axis (HPA). Suppression of HPA was shown in patients on long term use of morphine, chronic transdermal fentanyl, methadone, and tramadol [32, 33].

Moreover, patients with mu receptors polymorphism A118G would have more opioid endocrinopathy and more opioid suppression of neurons that release corticotropin releasing hormone (CRH) which would explain how opioids induced adrenal insufficiency in such individuals [34, 35].

## 5. Conclusion and Recommendations

Finally, it is concluded that chronic use of tramadol can cause many hazardous effects on different body organs. And, from this current study, it is approved that chronic use of tramadol caused adrenal insufficiency both histologically and biochemically and stoppage of tramadol use leads to a decrease in its hazardous effects not only on adrenal glands but also on different body organs.

So, it is recommended to address public associations and government agencies for raising awareness against hazards of tramadol. Holding seminars for students of preparatory and high schools should be done to obtain knowledge about bad effects of tramadol to reduce its addiction among them. Additionally, it is advised to add drug screening for tramadol to all forms of basic toxicological screening and to set strict sanctions on drivers and workers with a positive test and on pharmacies that sell tramadol without medical prescription. Finally, the results of our study emphasize the need for future researches that can explain the other possible mechanisms of adrenal insufficiency induced by chronic use of tramadol.

## Figures and Tables

**Table 1:** Details giving primer sequences, expected product sizes, and annealing temperature for the genes amplified.

Gene	Primer sequence (5-3)	Product size	Annealing temperature
GPX	Forward GGAGAATGGCAAGAATGAAGA	139 pb	55°C
	Reverse CCGCAGGAAGGTAAAGAG		
B-actin	Forward GAGAGGGAAATCGTGCGTGAC	452 pb	55°C
	Reverse CATCTGCTGGAAGGTGGACA		
Thioredoxin reductase	Forward CCGCAACAGCCAAAATGGTGA	339 pb	60°C
	Reverse AGCATGATTAGGCAAACTCCGTAA		
18s	Forward TTGACGGAAGGGCACCACCA	13 pb	55°C
	Reverse GCACCACCACCCACGGAATCG		

**Table 2:** One-way ANOVA statistical analysis of cortisol and DHEAS levels in different experimental groups (*n* = 10).

*DHEAS level (microgram/ml)*					*P value*
	Group				
	Group I	Group II	Group III	Group IV	
Mean ± SD	25.2 ± 2.1	13.53 ± 0.59	19.76 ± 0.89	23.5 ± 1.8	<0.001^*∗*^
Post hoc LSD analysis					
Group I		0.001^*∗*^	0.001^*∗*^	<0.001^*∗*^	
Group II			<0.001^*∗*^	<0.001^*∗*^	
Group III				<0.001^*∗*^	

*Cortisol level (nmol/L)*					*P value*
	Group				
	Group I	Group II	Group III	Group IV	

Mean ± SD	99.5 ± 1.3	63.42 ± 0.54	82.02 ± 1.09	95.4 ± 1.02	<0.001^*∗*^
Post hoc LSD analysis					
Group I		0.001^*∗*^	<0.001^*∗*^	<0.001^*∗*^	
Group II			<0.001^*∗*^	<0.001^*∗*^	
Group III				0.001^*∗*^	

^*∗*^ *P* < 0.05, significant; SD, standard deviation.

**Table 3:** One-way ANOVA statistical analysis of GSH-Px level in different experimental groups (*n* = 10).

	*GSH-Px level (mIU/ml)*				*P* value
	Group				
	Group I	Group II	Group III	Group IV	
Mean ± SD	212.14 ± 31.1	102.01 ± 21	172.3 ± 22.1	207.01 ± 26.2	<0.001^*∗*^
Post hoc LSD analysis					
Group I		0.001^*∗*^	0.001^*∗*^	<0.001^*∗*^	
Group II			<0.001^*∗*^	<0.001^*∗*^	
Group III				<0.001^*∗*^	

GSH-Px: glutathione peroxidase; ^*∗*^*P* < 0.05, significant; SD, standard deviation.

**Table 4:** One-way ANOVA statistical analysis of plasma TR level in different experimental groups (*n* = 10).

	*Plasma TR level (mIU/ml)*				*P* value
	Group				
	Group I	Group II	Group III	Group IV	
Mean ± SD	152.22 ± 22.1	82.27 ± 13.1	125.3 ± 14.3	148.08 ± 20.02	<0.001^*∗*^
Post hoc LSD analysis					
Group I		0.001^*∗*^	<0.001^*∗*^	<0.001^*∗*^	
Group II			<0.001^*∗*^	<0.001^*∗*^	
Group III				0.001^*∗*^	

TR: thioredoxin reductase; ^*∗*^*P* < 0.05, significant; SD, standard deviation.

**Table 5:** One-way ANOVA statistical analysis of MDA level in adrenal glands in different experimental groups (*n* = 10).

	*MDA level (µmol/mg wet tissue)*				*P* value
	Group				
	Group I	Group II	Group III	Group IV	
Mean ± SD	26.6 ± 8.1	62.1 ± 6.05	44.3 ± 11.3	30.8 ± 9.02	<0.001^*∗*^
Post hoc LSD analysis					
Group I		0.001^*∗*^	<0.001^*∗*^	0.001^*∗*^	
Group II			<0.001^*∗*^	<0.001^*∗*^	
Group III				<0.001^*∗*^	

^*∗*^ *P* < 0.05, significant; SD, standard deviation.

**Table 6:** One-way ANOVA statistical analysis of the intensity of the PCR product bands of GSH-Px among different experimental groups (*n* = 10).

	*Intensity of PCR product bands of GSH-Px*				*P* value
	Group				
	Group I	Group II	Group III	Group IV	
Mean ± SD	913.22 ± 31.1	412.74 ± 21	782.1 ± 22.1	908.01 ± 26.2	<0.001^*∗*^
Post hoc LSD analysis					
Group I		0.001^*∗*^	0.001^*∗*^	<0.001^*∗*^	
Group II			<0.001^*∗*^	<0.001^*∗*^	
Group III				<0.001^*∗*^	

GSH-Px: glutathione peroxidase; ^*∗*^*P* < 0.05, significant; SD, standard deviation.

**Table 7:** One-way ANOVA statistical analysis of the intensity of the PCR product bands of TR among different experimental groups (*n* = 10).

	*Intensity of PCR product bands of TR*				*P* value
	Group				
	Group I	Group II	Group III	Group IV	
Mean ± SD	820.12 ± 22.1	308.84 ± 15.2	682.04 ± 18.1	813.01 ± 21.2	<0.001^*∗*^
Post hoc LSD analysis					
Group I		0.001^*∗*^	0.001^*∗*^	0.001^*∗*^	
Group II			<0.001^*∗*^	<0.001^*∗*^	
Group III				0.001^*∗*^	

TR: thioredoxin reductase; ^*∗*^*P* < 0.05, significant; SD, standard deviation.
